# Dynamics and Complexity of Dark Fermentation Microbial Communities Producing Hydrogen From Sugar Beet Molasses in Continuously Operating Packed Bed Reactors

**DOI:** 10.3389/fmicb.2020.612344

**Published:** 2021-01-08

**Authors:** Anna Detman, Daniel Laubitz, Aleksandra Chojnacka, Ewa Wiktorowska-Sowa, Jan Piotrowski, Agnieszka Salamon, Wiktor Kaźmierczak, Mieczysław K. Błaszczyk, Albert Barberan, Yongjian Chen, Ewa Łupikasza, Fei Yang, Anna Sikora

**Affiliations:** ^1^Institute of Biochemistry and Biophysics, Polish Academy of Sciences, Warsaw, Poland; ^2^Department of Pediatrics, University of Arizona, Tucson, AZ, United States; ^3^Department of Biochemistry and Microbiology, Institute of Biology, Warsaw University of Life Sciences, Warsaw, Poland; ^4^Krajowa Spółka Cukrowa S.A. Production Facility Dobrzelin Sugar Factory, Dobrzelin, Poland; ^5^Institute of Agricultural and Food Biotechnology, Warsaw, Poland; ^6^Department of Environmental Science, University of Arizona, Tucson, AZ, United States; ^7^Faculty of Earth Sciences, University of Silesia in Katowice, Sosnowiec, Poland

**Keywords:** dark fermentation, anaerobic digestion, biohydrogen, microbial communities, lactic acid bacteria, hydrogen-producing bacteria, metagenomic analysis

## Abstract

This study describes the dynamics and complexity of microbial communities producing hydrogen-rich fermentation gas from sugar-beet molasses in five packed-bed reactors (PBRs). The bioreactors constitute a part of a system producing hydrogen from the by-products of the sugar-beet industry that has been operating continuously in one of the Polish sugar factories. PBRs with different working volumes, packing materials, construction and inocula were tested. This study focused on analysis (based on 16S rRNA profiling and shotgun metagenomics sequencing) of the microbial communities selected in the PBRs under the conditions of high (>100 cm^3^/g COD of molasses) and low (<50 cm^3^/g COD of molasses) efficiencies of hydrogen production. The stability and efficiency of the hydrogen production are determined by the composition of dark fermentation microbial communities. The most striking difference between the tested samples is the ratio of hydrogen producers to lactic acid bacteria. The highest efficiency of hydrogen production (130–160 cm^3^/g COD of molasses) was achieved at the ratios of HPB to LAB ≈ 4:2.5 or 2.5:1 as determined by 16S rRNA sequencing or shotgun metagenomics sequencing, respectively. The most abundant *Clostridium* species were *C. pasteurianum* and *C. tyrobutyricum.* A multiple predominance of LAB over HPB (3:1–4:1) or clostridia over LAB (5:1–60:1) results in decreased hydrogen production. Inhibition of hydrogen production was illustrated by overproduction of short chain fatty acids and ethanol. Furthermore, concentration of ethanol might be a relevant marker or factor promoting a metabolic shift in the DF bioreactors processing carbohydrates from hydrogen-yielding toward lactic acid fermentation or solventogenic pathways. The novelty of this study is identifying a community balance between hydrogen producers and lactic acid bacteria for stable hydrogen producing systems. The balance stems from long-term selection of hydrogen-producing microbial community, operating conditions such as bioreactor construction, packing material, hydraulic retention time and substrate concentration. This finding is confirmed by additional analysis of the proportions between HPB and LAB in dark fermentation bioreactors from other studies. The results contribute to the advance of knowledge in the area of relationships and nutritional interactions especially the cross-feeding of lactate between bacteria in dark fermentation microbial communities.

## Introduction

Hydrolysis and acidogenesis are the two initial steps of anaerobic digestion whereby complex organic matter is decomposed and processed mainly to short-chain fatty acids, alcohols, carbon dioxide and hydrogen, the identity and proportion of which depend on the type of fermentation. Dark fermentation (DF) is a part of the acidogenic step of anaerobic digestion which involves conversion of an organic substrate to hydrogen, carbon dioxide and non-gaseous products including acetic and butyric acids. DF is regarded as a promising alternative method of hydrogen (a pure energy carrier) production (Ghimire et al., 2015; Bharathiraja et al., 2016; Khan et al., 2016). Hydrogen is generated in glycolytic fermentations, i.e., clostridial-type and enterobacterial-type fermentation. Members of the order *Clostridiales*, the genera *Bacillus* and *Prevotella* and the *Enterobacteriaceae* family are well-recognized hydrogen producing bacteria (HPB). In the clostridial-type fermentation, pyruvate:ferredoxin oxidoreductase (PFOR) oxidizes pyruvate to acetyl-CoA in the presence of ferredoxin (Fd) that is simultaneously reduced. Also NADH:ferredoxin oxidoreductase (NFOR) can catalyze the reaction of ferredoxin reduction with NADH. Electrons from the reduced ferredoxin are used for proton reduction by hydrogenases and hydrogen is released. In the mixed acid-fermentation, pyruvate is converted to acetyl-CoA and formic acid by pyruvate formate-lyase (PFL). Formate hydrogen-lyase degrades the formic acid into hydrogen and carbon dioxide. In the *Enterobacter*-type fermentation, hydrogen can also be formed in the reactions of NADH oxidation by NFOR as described for the clostridial-type fermentation (Seppälä et al., 2011; Nasr et al., 2017; Maeda et al., 2018).

However, the processes in DF bioreactors are more complex and not only limited to the clostridial-type and enterobacterial-type fermentations. The acidogenic step of anaerobic digestion is a result of the metabolic activity and nutritional interactions between various groups of fermentative bacteria. Lactic acid bacteria (LAB) are a common component of the DF microbial community. The LAB are ubiquitous in the environment and, being part of the plant microflora, they enter with the biomass into anaerobic bioreactors. In a book chapter by Sikora et al. (2013), a provocative discussion has been undertaken on the relevance of the LAB in DF microbial communities and their impact on hydrogen producers. Many authors consider LAB as competitors for substrate that replace hydrogen-yielding fermentations with lactic acid fermentation and shut down hydrogen production. In the homolactic fermentation, two molecules of pyruvate are converted to two molecules of lactate whereas in the heterolactic fermentation the products are lactate, ethanol and carbon dioxide. The LAB can also secrete bacteriocins that inhibit the growth of the hydrogen-producing bacteria (Noike et al., 2002; Ren et al., 2007; Jo et al., 2008; Sreela-Or et al., 2011; Etchebehere et al., 2016; Palomo-Briones et al., 2018). On the other hand, lactate and acetate can be converted to butyrate, carbon dioxide and hydrogen. It is a nutritional interaction between lactate- and acetate-forming bacteria and butyrate producers (cross-feeding of lactate) recognized in the intestinal microbial community (Duncan et al., 2004; Belenguer et al., 2006, 2011; Moens et al., 2017), and also observed in DF bioreactors. It is also commonly known that many clostridia can use lactate and acetate for butyrate synthesis and hydrogen production. When grown on a medium where acetate and lactate were the exclusive carbon sources, *Clostridium acetobutylicum* (Diez-Gonzalez et al., 1995), *Butyribacterium methylotrophicum* (Shen et al., 1996), *Clostridium diolis* (Matsumoto and Nishimura, 2007), and *Clostridium tyrobutyricum* (Jo et al., 2008; Wu et al., 2012) produced butyrate, carbon dioxide and hydrogen. Also supplementation of fermentation substrates with lactic and acetic acids stimulated hydrogen production (Matsumoto and Nishimura, 2007; Jo et al., 2008; Baghchehsaraee et al., 2009; Kim et al., 2012). It is noteworthy that the conversion of lactate and acetate as a pathway of hydrogen production in DF bioreactors was originally proposed by Matsumoto and Nishimura (2007). Previously, using *Clostridium butyricum* as a new model, we have proposed an updated metabolic scheme of lactate and acetate conversion to butyrate (Detman et al., 2019). This scheme makes use of the current knowledge on the flavin-based electron bifurcation (Li et al., 2008; Weghoff et al., 2015; Peters et al., 2016). Briefly, a FAD-dependent lactate dehydrogenase LDH forms a stable complex with an LDH-specific electron-transfer flavoprotein (EtfA/B). According to the equation: 2NAD^+^ + Fd_red_ + lactate → 2NADH + Fd_ox_ + pyruvate, two simultaneous processes occur, i.e., (i) endergonic lactate oxidation to pyruvate using NAD^+^ as the oxidant and (ii) the oxidation of reduced ferredoxin (Weghoff et al., 2015). Pyruvate is converted to acetyl coenzyme A (acetyl-CoA) and the subsequent steps leading to butyrate formation are analogous to those of clostridial-type (butyric acid) fermentation with the requirement for an additional external acetate (Louis and Flint, 2009). The key reaction in the pathway of butyrate synthesis is the formation of butyryl-CoA described by the equation: 2NADH + Fd_ox_ + crotonyl-CoA → 2 NAD + Fd_red_ + butyryl-CoA. It is catalyzed by the butyryl-CoA dehydrogenase (Bcd)/Bcd-specific Etf complex (Bcd/EtfAB complex) and involves endergonic ferredoxin reduction with NADH coupled to exergonic crotonyl-CoA reduction with NADH (Li et al., 2008; Demmer et al., 2017; Buckel and Thauer, 2018).

The studies on fermentation of tequila vinasse, agave bagasse and nixtamalization wastewater under mesophilic conditions supplied a lot of data supporting the positive impact of cross-feeding of lactate on hydrogen production in DF bioreactors. Their authors postulate that lactate and acetate are the main substrates for hydrogen production since: (i) the highest efficiency was achieved by microbial communities composed of lactate, acetate and butyrate producers; (ii) a specific succession of bacteria was observed in batch experiments, where the substrate was firstly processed to acetate and lactate that were subsequently transformed to butyrate, hydrogen and carbon dioxide; (iii) pH was a relevant factor ensuring optimal conditions for the syntrophy between lactate- and butyrate- producers (García-Depraect et al., 2017, 2019a,b, 2019c; García-Depraect and León-Becerril, 2018). Moreover, the authors of these studies elaborated and patented a hydrogen-producing inoculum comprising acetate-, lactate- and butyrate-producing bacteria (American Type Culture Collection PTA 124566) (García-Depraect et al., 2019a).

The stimulating effect of cross-feeding of lactate on hydrogen synthesis was also observed in co-cultures of two bacterial strains: the butyrate- and hydrogen-producing *Clostridium beijerinckii* and the lactate-producer *Yokenella regensburgei* (Schwalm et al., 2019) or the hydrogen- and lactate-producing *Bacillus cereus* and the hydrogen- and butyrate-producing *Brevundimonas naejangsanensis* (Wang et al., 2019).

Conversion of lactate and acetate is a universal pathway of hydrogen production since it is also observed under thermophilic conditions. Studies on continuously operating bioreactors processing sugarcane vinasse at 55°C revealed that the pH determines the dominant metabolic pathways in the bioreactor. Hydrogen and butyrate production was observed at pH = 5.0–5.5. Positive correlations were found between LAB (dominated by *Lactobacillus* species), *Clostridium* species, butyrate and hydrogen. The thermophilic *Thermoanaerobacterium* genus was found as a relevant hydrogen-producer (Fuess et al., 2018, 2019).

Previously, we have also confirmed that DF microbial communities fed molasses are able to transform lactate and acetate to butyrate in batch experiments under mesophilic conditions (Detman et al., 2019). Then we have recognized the biodiversity and dynamics of DF microbial communities able and unable to convert lactate and acetate to butyrate, and the conditions for conversion. The relevant factors for transformation of lactate and acetate to butyrate in the presence of carbohydrates are pH in the range 5–6 and the balance between LAB (especially *Lactobacillus*), acetate producers (*Bifidobacterium)* and butyrate producers (mainly *Clostridium*) as well as *Prevotella* (Detman et al., 2020).

It is noteworthy that hydrogen production is governed by different factors such as the type of feedstock, the substrate concentration, the origin of inoculum, the hydrogen retention time (HRT), the minerals content, the pH conditions, the bioreactor construction that all influence microbial physiology and microbial interactions inside the DF microbial community.

The aim of the present study was to examine the structure of the DF microbial communities collected from well-performing and poorly performing PBRs to understand the relationships between bacteria in the microbial communities during processing of molasses to hydrogen, and to find key players and factors related to high and low efficiency of hydrogen production.

## Materials and Methods

### Experimental Set-Up for Continuous Hydrogen Production

A two-stage system for continuous hydrogen and methane production from molasses operating in the Dobrzelin Sugar Factory, a branch of the Polish Sugar Company “Polski Cukier,” was described previously (Detman et al., 2017). Here, some modifications to the stage 1 of the system, the hydrogen-producing bioreactors, were introduced. The modifications concerned working volume, packing material, construction, material design, position of the bioreactor (vertical vs. horizontal) as well as operating conditions. There were five PBRs made of stainless steel: four of them filled with slag as the packing material, with the working volume of 11.5 L, put vertically, designated as PBR1 and PBR2, or horizontally, designated as PBR3, and with the working volume of 12 L, put horizontally, designated as PBR4; the fifth one - without packing material, with the working volume of 32 L, put vertically, designated as PBR5. The cultivation medium was 10-fold diluted M9 medium (Miller, 1972) supplemented with molasses from the sugar factory. The concentration of molasses was 10, 20, 40 g/L corresponding to 8.5, 17, and 34 g COD (chemical oxygen demand) per L, respectively. The hydraulic retention times (HRT) were 5–31 h (Table 1). The inoculum for PBR2 through PBR5 was an anaerobic sludge from the waste water treatment plant in the Dobrzelin Sugar Factory subjected to a heat treatment. The inoculum for PBR1 was the microbial community described previously that originally came from the bottom sediment of a meromictic lake (Chojnacka et al., 2011; Detman et al., 2017) enriched with the heat-treated anaerobic sludge from the waste water treatment plant.

**TABLE 1:** Parameters describing the performance of PBRs at selected time points.

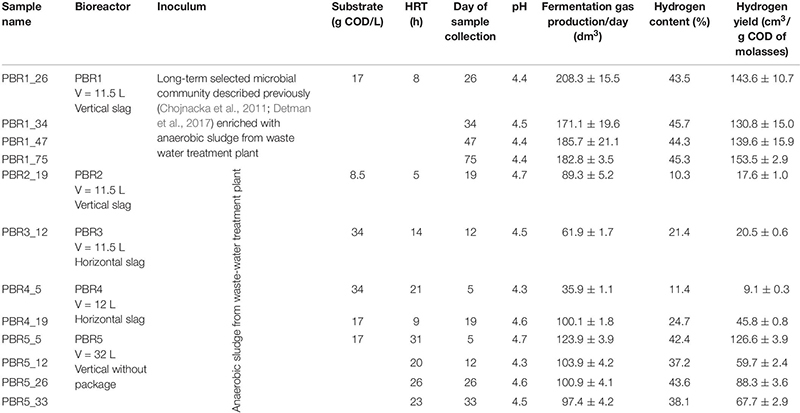

**FIGURE 1:**
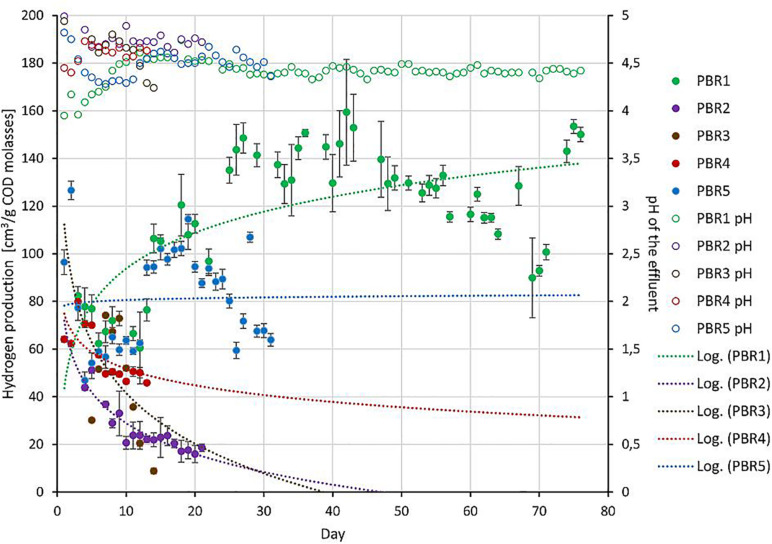
The long-term performance of PBRs tested in this study: hydrogen production (solid circles, left axis) and pH of the effluents (open circles, right axis) from bioreactors. Since the operation periods were different, trend lines were added for hydrogen-production data sets.

*Long-term performance is shown in Figure 1.*

The naming of the samples is as follows: PBR number_day of operation, e.g., PBR1_26 designates the sample collected from PBR1 on day 26; PBR2_10 designates the sample collected from PBR2 on day 10.

### Analytical Methods

The total rate of gas production from the PBRs was measured (10 measurements for each time point, for each PBR) using a bubble flowmeter (Zakłady Urządzeń Przemysłowych ZAM Kęty, Poland). In each case a mean ± SD (standard deviation) was calculated. The composition of the fermentation gas was analyzed by GC/TCD (gas chromatography with thermal conductivity detector) using an Agilent Technologies model 7890B gas chromatograph. The pH of the media and the digestion liquids was measured using a standard pH meter (ELMETRON model CP-502, Poland). The XLSTAT by Addinsoft was used to calculate Kolmogorov–Smirnov and Mann–Whitney tests for detailed presentation of hydrogen production over time and testing similarities between the measurement series for each bioreactor. All the samples were centrifuged (7000 × *g* for 10 min, 10°C) to remove microbial cells and debris, and the COD, the concentrations of carbohydrates (sucrose, glucose, and fructose), short-chain fatty acids, and ethanol were determined. The COD and the concentrations were determined in the supernatants obtained after centrifugation of the digestive liquids as described above. The COD was determined using a NANOCOLOR COD 1500 kit (Machery-Nagel) according to ISO 1575:2002.

The carbohydrates and ethanol were analyzed using high performance liquid chromatography (HPLC) with refractometric detection (Waters HPLC system: Waters 2695 – Separations Module, Waters 2414 – Refractive Index Detector, a thermostat for column, and 300 × 6.5 mm Sugar Pak I column with guard column). The quantification of the carbohydrates was carried out at 90°C, and of the ethanol at 70°C. The sample (10 μL) was injected into the column and eluted for 20 min with an isocratic flow of 0.1 mM calcium disodium salt of EDTA (0.5 mL/min).

Short-chain fatty acids were analyzed by HPLC with photometric detection (Waters HPLC system as above, Waters 2996 – Photodiode Array Detector, and 300 × 7.8 mm Aminex HPX-87 H column with guard column at 30°C). The samples were eluted for 45 min with an isocratic flow (0.6 mL/min) of 4 mM sulfuric acid.

### Microbial DNA Isolation

Two 30-ml samples (duplicates) of the microbial community were collected from each bioreactor (PBR1–PBR5) at selected days as shown in Table 1: days 26, 34, 47, and 75 from PBR1; day 19 from PBR2; day 12 from PBR3; days 5 and 19 from PBR4; days 5, 12, 26, and 33 from PBR5. The samples came from the inside middle part of each bioreactor and contained granular structures suspended in fluid phase. The total DNA was isolated from the pellets obtained after centrifugation (see above) of 2 ml-samples of the microbial communities. For each bioreactor the DNA was isolated from two samples (duplicates). DNA was extracted and purified using a DNeasy PowerSoil Pro Kit (Qiagen, Cat. No. 47014) according to the manufacturer’s protocol. Cell lysis was done using Vortex-Genie 2 equipped with a Vortex Adapter for 1.5–2 ml tubes (cat. no. 13000-V1-24). DNA was stored at −20 °C. The final samples of DNA extracted from the two duplicates were pooled.

### 16S rRNA Sequencing and Data Analysis

The hypervariable V4 region of the 16S rRNA gene was amplified from each sample using barcoded reverse primers (806R) unique for each sample and a common forward primer (515F). Both the reverse and the forward primers were extended with the sequencing primer pads, linkers, and Illumina adapters (Caporaso et al., 2012). The PCR was performed using MyFi^TM^ Mix (Bioline Meridian, Cat. No. BIO-25050) on LightCycler 96 (Roche) in a final volume of 40 μL. Amplicons were quantified using Quant-It PicoGreen dsDNA Assay kit (Thermo Fisher Scientific, Cat. No. P7589), according to the manufacturer’s protocol. Equal amounts of amplified DNA (240 ng) from each sample were pooled into a sequencing library followed by removing DNA fragments smaller than 120 bp (unused primers and dimer primers) with UltraClean PCR Clean-Up Kit (MoBio, Cat. No. 12500). The final amplicon concentration was quantified by qPCR with KAPA Library Quantification Kit for Illumina Platforms (KAPA Biosystems, Cat. No. KK4854) in the presence of a set of six DNA standards (KAPA Biosystems, Cat. No. KK4905). Subsequently, the library was diluted to a concentration of 4nM, and denatured with 0.1N NaOH. The library was sequenced at the Microbiome Core at the Steele Children’s Research Center, University of Arizona, using MiSeq platform (Illumina) and custom primers. Due to the limited sequence diversity among 16S rRNA amplicons, 5% of the PhiX Sequencing Control V3 (Illumina, Cat. No. FC-110-3001) was used to spike the library to increase diversity. The raw sequencing data were demultiplexed and barcodes trimmed using *idemp* script^1^. Filtering, dereplication (grouping all identical sequences into unique sequence variants), chimera identification and removal, merging paired-end reads, and creating an Amplicon Sequence Variant (ASV) table were performed with DADA2 R package (Callahan et al., 2016). The taxonomy to the ASVs was assigned using the Ribosomal Database Project (RDP) classifier (Wang et al., 2007) against the SILVA database release 132 (Quast et al., 2013).

The Alpha Diversity measures, including ASVs abundance (Richness), and biodiversity indices (Shannon and Simpson indices), were calculated. The differences in microbial communities were evaluated using non-metric multidimensional scaling (NMDS) ordination analysis based on Bray–Curtis distances. Also, to investigate and visualize the association between metadata variables and their effect on the species distribution pattern, redundancy analysis was performed using the *vegan* R package (Oksanen et al., 2019). The results obtained were visualized with the *ggplot2* package (ver. 3.3.2^2^) (Wickham, 2016) and with the *Heatplus* (ver. 3.11) R package (Ploner, 2020).

The raw sequences generated in this study have been deposited in NCBI databases with the accession number PRJNA645198 (submission number: SUB8107482).

### Shotgun Metagenomics Sequencing and Data Analyses

The libraries for metagenomics were constructed for samples selected from PBR1, PBR4, and PBR5 using QIASeq FX DNA Library Kit (QIAGEN) according to the manufacturer’s protocol. Briefly, 50 ng of DNA from each sample (or pooled samples) was randomly fragmented with FX Enzyme Mix followed by the adapter ligation step. Both i5 and i7 adapters contain unique 8-nucleotide barcodes. After removing free adapters from the reaction with AMPure XP magnetic beads, all individual libraries were amplified by PCR followed by the size selection with 2-step purification (the negative selection followed by the positive selection step) with AMPure XP magnetic beads. The quality and quantity of all libraries was determined with Agilent 4150 TapeStation DNA analyzer. The libraries were normalized and pooled, and the sequencing was performed on the Illumina NextSeq 500/550 platform using Illumina 400M HighOutput 300-cycles sequencing chemistry. Adapter sequences were removed using Cutadapt v. 2.1 (Martin, 2011). Reads shorter than 50 bp and low-quality bases were removed using Trimmomatic v. 0.38 (Bolger et al., 2014). The high-quality reads were *de novo* assembled using Megahit ver. 1.1.4 (Li et al., 2015). After discarding assembled contigs shorter than 500 bp, protein-coding genes were predicted using Prodigal v. 2.6 (Hyatt et al., 2010). Paired-end reads were mapped to the genes using BWA ver. 0.7.16 (Li and Durbin, 2010). The raw sequences generated in this study have been deposited in NCBI databases with the accession number PRJNA661691.

Functional information of the genes was annotated by comparison to the Kyoto Encyclopedia of Genes and Genomes (KEGG) database using GhostKOALA^3^ (Kanehisa et al., 2016).

## Results

### Hydrogen Production in PBRs Under Different Operating Conditions

Packed-bed reactors, the object of this study, are a part of the two-stage installation producing hydrogen and methane from molasses under mesophilic conditions in the Dobrzelin Sugar Factory (Krajowa Spółka Cukrowa S.A., Poland). The installation was described previously (Detman et al., 2017) and it has been still operating to understand the mechanisms of hydrogen and methane production, to optimize and scale up the process. Here we focus on the hydrogen production step of the installation. Figure 1 presents the hydrogen yield obtained during continuous operation of five bioreactors under the different operating conditions tested. Since the operation periods were different, the trend lines were added for each data set. The operation periods differed because only promising variants (such as PBR1) were maintained for a long time due to economic reasons. Additionally, Table 1 shows detailed parameters describing the PBRs’ performance near the points in time when samples were taken for the biodiversity analysis presented in Section “Biodiversity of DF Microbial Communities and Metabolic Activities.”

The bioreactors were divided into three groups depending on the efficiency of hydrogen production: >100 cm^3^/g COD of molasses (PBR1), 50–100 cm^3^/g COD of molasses (PBR5), <50 cm^3^/g COD of molasses (PBR2, PBR3, PBR4) (Figure 1 and Table 1).

Under the optimal conditions the hydrogen yield was maintained at over 100 cm^3^/g COD of molasses and reached even 150 cm^3^/g COD of molasses (PBR1 in Figure 1 and Table 1). The optimal conditions included a vertical bioreactor with a working volume of 11.5 L filled with slag; substrate concentration 17 g COD of molasses/L, HRT = 8 h, a concentration of KH_2_PO_4_, NH_4_Cl, NaCl, MgCl_2_, CaCl_2_ corresponding to a 10-fold diluted M9 medium. The inoculum was the long-term-selected microbial community described previously (Chojnacka et al., 2011; Detman et al., 2017) enriched with the anaerobic sludge from the waste water treatment plant. This inoculum turned out to be the most effective. Stability of hydrogen production in PBR1 was confirmed by Kolmogorov–Smirnov and Mann–Whitney tests. The overtime comparison of data series between subsequent time points shows either no statistically significant differences or diminishing significance level (*p*-value) of variations between the samples (Supplementary Table 1).

Hydrogen efficiency ≤ 50 cm^3^/g COD of molasses was observed for three bioreactors with a working volume of ∼12 L, filled with slag, inoculated with the anaerobic sludge from the waste water treatment plant: vertical PBR2, horizontal PBR3 and PBR4, operating at different HRT (5 h for PBR2, 14 h for PBR3, 9 and 21 h for PBR4) and a modified substrate concentration (8.5 g COD of molasses/L for PBR2, 17 g COD of molasses/L for PBR4, 34 g COD of molasses/L for PBR3 and PBR4). The overtime comparison of data series between subsequent time points for PBR2 shows stable low hydrogen production ∼20 cm^3^/g COD of molasses starting from the sample PBR2_10 (most of comparisons without statistically significant differences, Kolmogorov–Smirnov and Mann–Whitney, tests *p* > 0.05). The sample PBR2_19 collected for biodiversity analysis well represents this state of PBR2 operation (Supplementary Table 2). Instability of hydrogen production in PBR3 was confirmed by the overtime comparison of data series between subsequent time points during the whole bioreactor operation (*p* = 0.0001) (Supplementary Table 3). PBR4 produced hydrogen at a rate of ≤50 cm^3^/g COD of molasses starting from the sample PBR4_13 where the degree of variations between the samples is decreasing (Supplementary Table 4). Sample PBR4_19 for biodiversity analysis was collected at the end of the PBR4 operation.

Efficiency of hydrogen production in the range of 50–100 cm^3^/g COD of molasses was observed for the vertical PBR5 with a working volume of 32 L without packing, processing molasses at the concentration corresponding to 17 g COD/L, at HRT 23–31 h. Interestingly, a short-term hydrogen production >100 cm^3^/g COD of molasses was also reported at the beginning of PBR5 operation. The overtime comparison of data series between subsequent time points shows only a short period between PBR5_16 and PBR_20 of stable hydrogen production (no statistically significant differences). The samples collected for biodiversity analysis PBR5_5, PBR5_12, PBR5_26 and PBR5_33 show statistically significant differences in hydrogen production (*p* < 0.0001) (Supplementary Table 5).

### Biodiversity of DF Microbial Communities and Metabolic Activities

**FIGURE 2:**
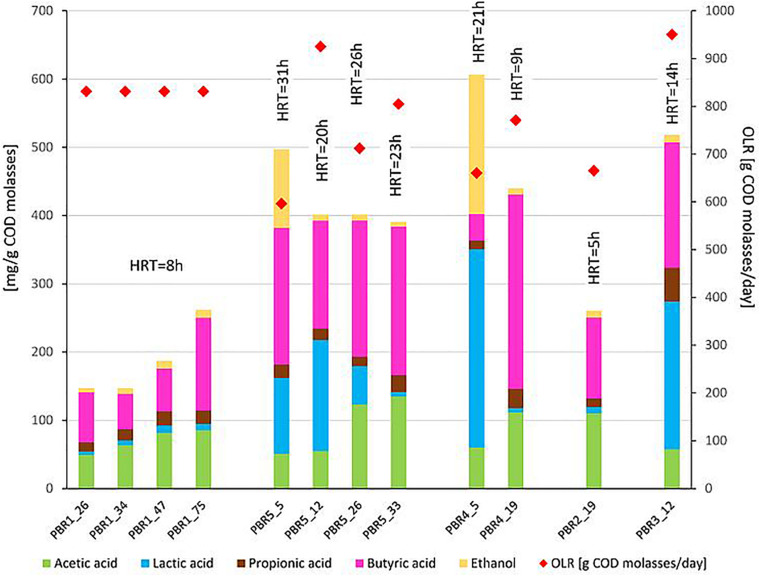
Non-gaseous fermentation products expressed in mg per g of COD of molasses (bars, left axis) and substrate concentration in g of COD of molasses per day (diamonds, right axis) supplied to the bioreactors in selected time points of operation. The composition of fermentation products was analyzed using high-performance liquid chromatography. The data comes from two parallel measurements. HRT is shown over each bar.

**FIGURE 3:**
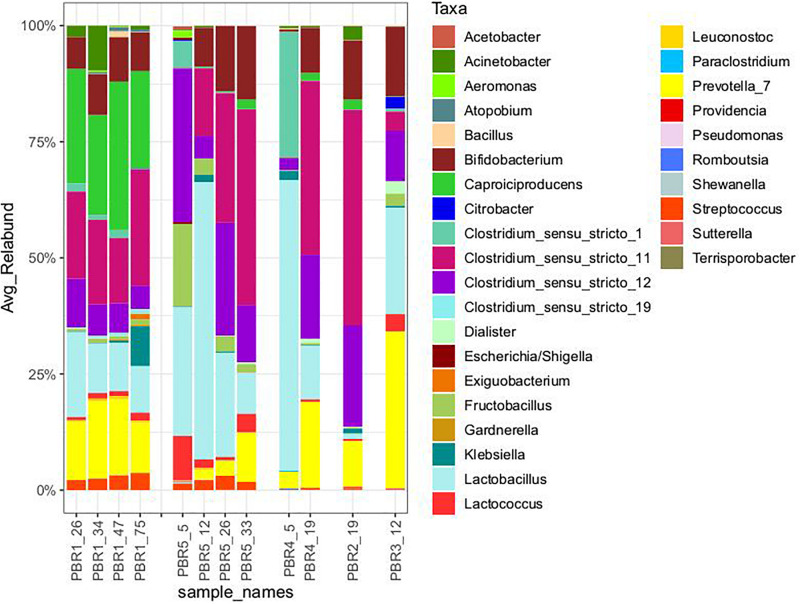
Taxonomic composition (genus level) of the microbial communities that selected out in PBRs, based on hypervariable V4 region of the 16S rRNA gene, sequenced on MiSeq platform (Illumina). The taxonomy was assigned using RDP classifier against the SILVA database. All taxa with Relative Abundance lower than 0.1% were removed.

**FIGURE 4:**
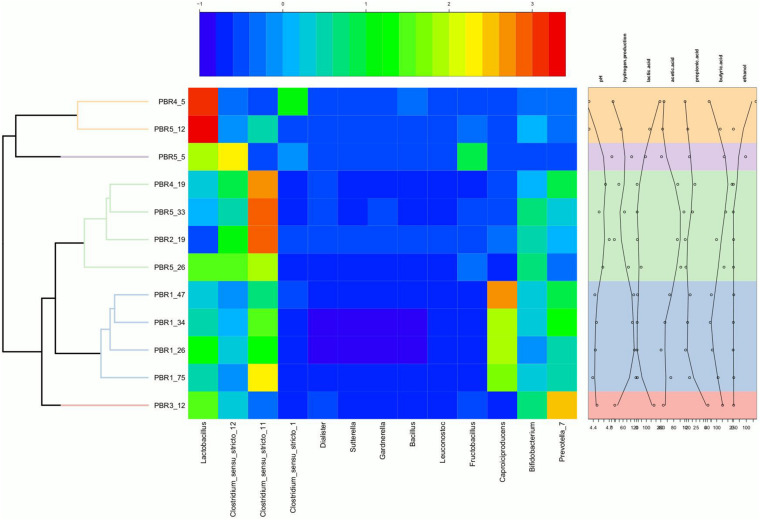
Heatmap showing the relative abundance of genera in the individual PBRs in selected time points with annotation of measured metabolites and pH. The heatmap was generated in R (Heatplus package, annHeatmap2 function) using the relative abundance of the observed genera. For clarity, all genera with summarized relative abundance lower than 0.1% were removed. Rows were clustered using average-linkage hierarchical clustering based on the Bray–Curtis dissimilarity matrix of the dataset (‘vegdist’ from the vegan package).

Hydrogen production was analyzed in relation to the non-gaseous fermentation products and taxonomic composition of the microbial communities (Figures 2–4). Note that Figures 2, 4, similarly to Table 1, show data near the points in time when samples were taken for biodiversity analysis. Microbial communities in all bioreactors were analyzed by sequencing of the V4 fragment of the gene encoding 16S rRNA (Figure 3). For detailed taxonomic assignments see Supplementary Table 6. All samples for this study were sequenced together with another project. For this study, the number of reads per sample was from 40,388 to 255,181. After quality control filtering and removing chimeras, the number of retained reads per samples was 35,348 to 213,929, respectively. The blank control (an empty sample processed as all other samples including the DNA extraction step) was sequenced along with all other samples and was removed during the rarefaction step due to a very low number of reads. To even sampling depth, the samples were rarefied at 35,348 reads.

With the most stable hydrogen production above 100 cm^3^/g COD of molasses (130–150 cm^3^/g COD of molasses) in PBR1, lactic acid concentrations in the effluents did not exceed 11 mg/g COD of molasses and butyric acid concentrations were in the range of 52–135 mg/g COD of molasses (PBR1, samples PBR1_26, PBR1_34, PBR1_47, PBR1_75). The microbial communities most stably producing hydrogen were composed of *Clostridium*_sensu_stricto_11 (14–25%), *Clostridium*_sensu_stricto_12 (5–11%), *Caproiciproducens* (21–32%), *Prevotella* (11–16%), *Bifidobacterium* (7–9%), *Acinetobacter* (1–9%), LAB, especially *Lactobacillus* (10–18%), *Streptococcus* (2–4%) and *Lactococcus* (1–2%). The minor species (<1%) were *Aeromonas*, *Atopobium, Bacillus, Citrobacter*, *Clostridium*_sensu_stricto_1, *Clostridium*_sensu_stricto_19, *Dialister, Exiguobacterium, Fructobacillus, Gardnerella, Klebsiella* (except sample PBR1_75) and *Leuconostoc.*

In the case of the short-term performance of PBR5 with hydrogen production equaling 127 cm^3^/g COD of molasses (sample PBR5_5), LAB were more abundant in comparison to the PBR1 with the contribution of *Lactobacillu*s (27.7%), *Fructobacillus* (17.9%), *Lactococcus* (9.3%) and *Streptococcus* (1.5%). *Clostridium*_sensu_stricto_12, *Clostridium*_sensu_stricto_1 and *Clostridium*_sensu_stricto_11 constituted 32.9%, 5.8% and 0.2%, respectively. The minor genera (<1%) were *Acetobacter, Acinetobacter, Aeromonas, Bifidobacterium, Citrobacter, Escherichia/Shigella, Klebsiella, Providencia* and *Shewanella*. Interestingly, in the sample PBR5_5 a relatively high concentration of ethanol, 115 mg/g COD of molasses, was detected in comparison to the samples from PBR1 where the corresponding values did not exceed 12 mg/g COD of molasses. Also the concentration of lactic acid was 10-fold higher compared with PBR1. After a few days, the PBR5 shifted from hydrogen production above 100 cm^3^/g COD of molasses (sample PBR5_5) to hydrogen production in the range 50–90 cm^3^/g COD of molasses (samples PBR5_12, PBR5_26, PBR5_33) due to changes in the metabolic activity and the structure of the microbial community. This indicates instability of the processes in a bioreactor with no packing material. In comparison to the sample PBR5_5 (day 5), the sample PBR5_12 (day 12) was dominated by LAB (*Lactobacillus*, 60%; *Bifidobacterium*, 8.4%; *Fructobacillus*, 3.4%; *Lactococcus*, 1.9%; *Streptococcus*, 2.1%) and a high concentration of lactic acid (162 mg/g COD of molasses) was detected in the effluents from the PBR5 (PBR5_12). The contribution of *Clostridium*_sensu_stricto_1 and *Clostridium*_sensu_stricto_12 decreased to 0.25% and 4.9%, respectively, whereas *Clostridium*_sensu_stricto_11 increased to 14.5%. In the samples PBR5_26 (day 26) and PBR5_33 (day 33) in comparison to the sample PBR5_12, decreases in the contribution of *Lactobacillus* (22.3% and 8.8%, respectively) and lactic acid concentration (56 and 7 mg/g COD molasses) with simultaneous increases of *Clostridium*_sensu_stricto_11 (27.8% and 42.0%, respectively) and *Clostridium*_sensu_stricto_12 (24.4% and 12.5%, respectively) as well as *Prevotella* (10.6% in sample PBR5_33) were observed. The concentration of acetic acid was relatively high (above 120 mg/g COD of molasses) both on days 26 (sample PBR5_26) and 33 (sample PBR5_33) probably due to a high contribution of *Bifidobacterium* in the microbial community (14% and 16% in samples PBR5_26 and PBR5_33, respectively). In all the samples from PBR5 butyric acid was a dominant component.

The next samples collected from PBR2, PBR3, and PBR4 represent the group of hydrogen production below 50 cm^3^/g COD of molasses. The effluent from PBR4 fed molasses at a concentration of 34 g COD/L (sample PBR4_5) was characterized by a high concentration of lactic acid (290 mg/g COD of molasses) and ethanol (205 mg/g COD of molasses). The concentration of acetic and propionic acids were 60 and 12 mg/g COD of molasses, respectively, whereas the concentration of butyric acid was relatively low (38 mg/g COD of molasses). The microbial community was dominated by *Lactobacillus* (62.5%), *Clostridium*_sensu_stricto_1 (27.1%), *Prevotella* (3.5%) and *Clostridium*_sensu_stricto_12 (2.6%). The minor genera (<1%) were *Acinetobacter, Bacillus, Bifidobacterium, Clostridium*_sensu_stricto_11, *Fructobacillus, Klebsiella, Paraclostridium, Romboutsia* and *Terrisporobacter*. The bioreactor (sample PBR4_5) produced hydrogen at a rate of 9.1 cm^3^/g COD of molasses. A two-fold decrease of molasses concentration in the medium caused dramatic changes in the metabolic activity in PBR4 (sample PBR4_19). A 7-fold increase in butyric acid concentration (to 284 mg/g COD of molasses), a 2-fold increase of acetic and propionic acids (112 and 28 mg/g COD of molasses, respectively), a 23-fold decrease of ethanol (to 9 mg/g COD of molasses) and a 48-fold decrease of lactic acid (to 6 mg/g COD of molasses) were observed. The hydrogen yield increased five-fold to 45.8 cm^3^/g COD of molasses, however, it did not achieve the values observed for PBR1 and PBR5. Significant changes were also observed in the structure of the PBR4 microbial community (sample PBR4_19) that was composed of *Clostridium*_sensu_stricto_11 (37.5%, 220-fold increase), *Clostridium*_sensu_stricto_12 (18.2%, 7-fold increase), *Prevotella* (18.4%, 5-fold increase), *Lactobacillus* (11.6%, 5-fold decrease), *Bifidobacterium* (9.7%, 16-fold increase), *Caproiciproducens* (1.6%). The minor genera (<1%) were *Acetobacter, Acinetobacter, Dialister, Fructobacillus, Klebsiella, Lactococcus, Streptococcus* and *Sutterella.* The microbial community that selected out in PBR2 (sample PBR2_19) fed a molasses-containing medium at the lowest concentration of 8.5 g COD of molasses/L was dominated by *Clostridium*_sensu_stricto_11 (46.5%), *Clostridium*_sensu_stricto_12 (21.8%), *Prevotella* (9.8%), *Bifidobacterium* (12.8%), *Acinetobacter* (2.8%), *Caproiciproducens* (2.1%), *Lactobacillus* (1.2%) and *Klebsiella* (1.0%). The minor genera (<1%) were *Bacillus, Dialister, Fructobacillus, Lactococcus, Streptococcus* and *Sutterella*. It is noteworthy that the contribution of LAB in the AS115 microbial community was the lowest in all samples tested. The composition of the effluent from PBR2 reflects the structure of the microbial community. A low concentration of lactic acid and ethanol, 9 and 10 mg/g COD of molasses, respectively, and a relatively high concentration of acetic and butyric acids, 110 and 118 mg/g COD of molasses, respectively, were detected. Hydrogen was produced at a rate of 17.6 cm^3^/g COD of molasses.

The last bioreactor from this group (PBR3, sample PBR3_12) was fed a molasses-containing medium at the concentration of 34 g COD of molasses/L and directed horizontally. Relatively high concentrations of non-gaseous fermentation products, especially lactic acid (217 mg/g COD of molasses), butyric acid (183 mg/g COD of molasses) and propionic acid (49 mg/g COD of molasses), were detected. The microbial community was composed of butyrate and lactate producers. The dominant genera were *Prevotella*_7 (33.7%), *Lactobacillus* (23%), *Bifidobacterium* (14.9%), *Clostridium_*sensu_stricto_12 (11%), *Clostridium*_sensu_stricto_11 (4.1%), *Lactococcus* (3.7%), *Fructobacillus* (2.7%), *Citrobacter* (2.7%) and *Dialister* (2.6%). *Bacillus*, *Clostridium*_sensu_stricto_1, *Klebsiella* and *Sutterella* were the minor genera (<1%). PBR3 produced hydrogen at a rate of 20.5 cm^3^/g COD of molasses.

The above results are summarized in Table 2 by calculation of the proportions between potential HPB (genera *Clostridia* sensu stricto and *Prevotella*), LAB and *Caproiciproducens* in the microbial communities. The issue of the *Caproiciproducens* genus is ambiguous and will be discussed further. Table 2 presents also the proportions between genera *Clostridia* sensu stricto and *Lactobacillus.* The proportions between the selected groups of bacteria were combined with the results of hydrogen production efficiency. In the most stably hydrogen producing (above 100 cm^3^/g COD of molasses) PBR1 filled with a packing bed, the ratio HPB:LAB:*Caproiciproducens* was 4:2.5:2.5, whereas the ratio genera *Clostridia* sensu stricto*:Lactobacillus* was 2.5:1. In PBR5 without packing bed the optimal ratios for hydrogen production were 4:5.5:0 and 4:3 for HPB:LAB:*Caproiciproducens* and genera *Clostridia* sensu stricto:*Lactobacillus*, respectively. However, the PBR5_5 microbial community occurred to be unstable and changed in time to the prevalence of LAB with increasing concentration of lactic acid among the fermentation products or to the prevalence of clostridia with increasing concentration of butyric acid among the fermentation products. All the changes in the proportions between the selected groups of bacteria resulted in a two-fold decrease in hydrogen production. The same tendency was observed in PBR2 and PBR4 (predominance of *Clostridia* sensu stricto in PBR2_19 and PBR4_19; predominance of LAB in PBR4_5). PBR3_12 is a specific sample, where the ratio of potential HPB:LAB was almost 1:1 and high concentrations of both lactic and butyric acids were detected among fermentation products. Probably it was due to the relatively high concentration of molasses (34 g COD/L) and the horizontal position of the bioreactor.

**TABLE 2:** Proportions between potential HPB:*Clostridia* sensu stricto, *Prevotella*, lactic acid bacteria and *Caproiciproducens* in the PBR1–PBR5 microbial communities based on 16S rRNA amplicon sequences, and the corresponding hydrogen production results.

Sample/bioreactor	Hydrogen production (cm^3^/g COD molasses)	HPB:LAB: *Caproiciproducens* ratio	*Clostridia* sensu stricto:*Lactobacillus* ratio
PBR1_26	130–160	4.5:3:2.5	2:1
PBR1_34		2:1:1	2.5:1
PBR1_47		4:2.5:3	2.5:1
PBR1_75		4:2.5:2	3:1
PBR5_5	∼130	4:5.5:0	4:3
PBR5_12	60–90	1:3:0	1:3
PBR5_26		5.5:4.5:0	5:2
PBR5_33		2:1:0	6:1
PBR2_19	∼20	5:1:0	60:1
PBR3_12	∼20	5:4.5:0	2:3
PBR4_5	∼10	1:2:0	1:2
PBR4_19	∼50	3:1:0	5:1

**FIGURE 5:**
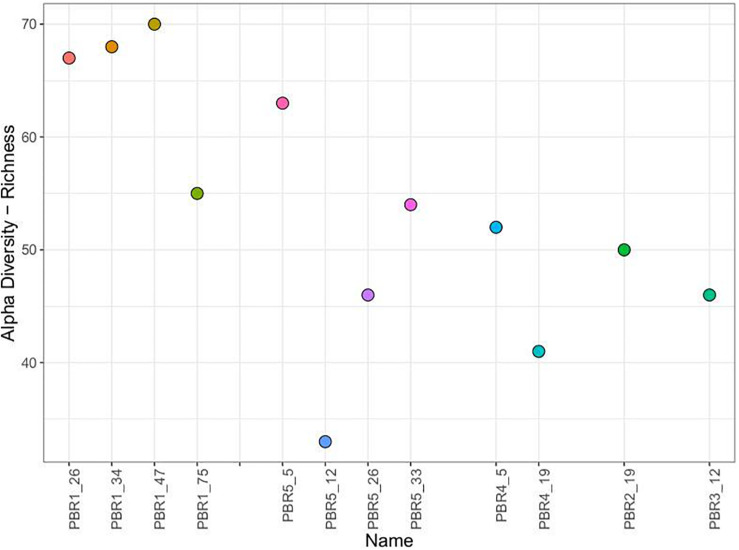
Alpha diversity (richness) of the microbial communities that selected out in PBRs.

Furthermore, the microbial communities (samples PBR1_26, PBR1_34, PBR1_47, PBR1_75) sampled from the PBR1 producing hydrogen most stably showed the highest biodiversity measured by taxonomic richness (Figure 5) and evenness (Shannon and Simpson indices) (Supplementary Figure 1).

### RDA Analysis

**FIGURE 6:**
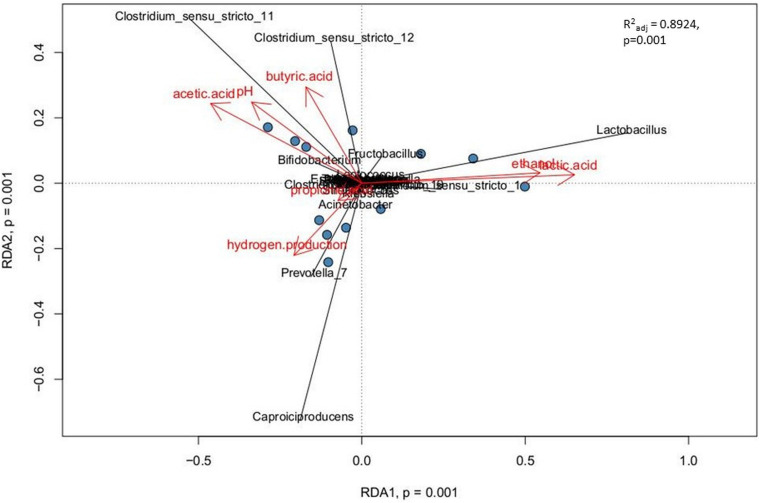
Correlations between the non-gaseous fermentation products, pH of the effluents from bioreactors and the dominant bacterial taxa presented as canonical Redundancy Analysis (RDA) for PBR1 through PBR5. Permutation tests (ANOVA) for RDA, for individual axes, and adjusted coefficients of determination *R*^2^adj were calculated with the *vegan* package.

Relationships between explanatory variables (the fermentation products, pH of the effluents) and genera with relative abundance at least 1% were visualized by using a redundancy analysis (RDA) on non-rarified center-log-ratio genera count tables. The RDA integrates the targeted metabolomic data with the analyses of sample biodiversity in all bioreactors. This model explains 89.2% of the variation in the data (*R*^2^_adj_ = 0.8924). The adjusted *R*^2^ measures the unbiased amount of explained variation. The analysis presents statistically significant correlations (*p* = 0.001, ANOVA permutation test) (Figure 6).

The following three positive correlations were observed: (i) C*lostridium*_sensu_stricto_12 and C*lostridium*_sensu_stricto_11 with butyric acid, acetic acid and pH; (ii) *Lactobacillus* with ethanol and lactic acid; (iii) *Caproiciproducens* and *Prevotella_*7 with hydrogen production. The correlation between *Caproiciproducens* and hydrogen production was unexpected and required further explanation by using metagenomic analysis.

### Metagenomic Analysis

For better recognition of the microbial communities on the genus and species level and to elucidate their metabolic potential, we selected four samples from the PBRs and subjected them to the shotgun metagenomics analysis. The samples were from (i) PBRs producing more than 100 cm^3^ hydrogen/g COD of molasses, designated as PBR1_26, PBR1_75 and PBR5_5 and (ii) PBRs producing less than 50 cm^3^ hydrogen/g COD of molasses, designated as PBR4_19 and PBR5_12. Relative abundances of dominant phyla derived from metagenomic sequencing were significantly correlated with those derived from 16S rRNA amplicon sequencing (Supplementary Figure 3).

A total of 94,487,950; 140,758,544; 140,988,916; 109,407,670 and 166,237,688 reads per sample were obtained for PBR1_26, PBR1_75, PBR5_5, PBR5_12 and PBR4_19, respectively. For detailed taxonomic composition of the microbial communities on the level of phylum, class, family and genus see the Supplementary Figure 2 and Supplementary Table 7.

In all samples the most abundant phylum was *Firmicutes* followed by *Actinobacteria, Bacteroidetes* and *Proteobacteria.* The *Firmicutes* were mainly represented by the *Bacilli* and *Clostridia* classes. On the level of family all the samples were composed mainly of *Clostridiaceae* (with the genus *Clostridium*), *Lactobacillaceae* (with the genus *Lactobacillus*); less numerous were *Bifidobacteriaceae* (with the genus *Bifidobacterium*), *Leuconostocaceae* (with the genus *Leuconostoc*), *Prevotellaceae* (with the genus *Prevotella*), *Streptococcaceae* (with the genera *Streptococcus, Lactococcus*), *Enterobacteriaceae, Lachnospiraceae, Peptostreptococcaceae* and *Ruminococcaceae*. The metagenomic analysis confirmed the results obtained by 16S rRNA sequencing. The most striking difference between the tested samples is the ratio of hydrogen producers (especially *Clostridium, Ruminococcus, Prevotella*) to LAB (especially *Lactobacillus, Bifidobacterium, Leuconostoc, Streptococcus, Lactococcus*) in the microbial communities. The difference is closely related to the hydrogen production efficiency. In the PBRs with high efficiency of hydrogen production (above 100 cm^3^/g COD of molasses) the ratio was approximately 2:1 in PBR1 (samples PBR1_26, PBR1_75) or 1:2 in PBR5 (PBR5_5). In the PBRs with low efficiency of hydrogen production (below 50 cm^3^/g COD of molasses) the ratio was 1:4 in PBR5 (sample PBR5_12) or 5:1 in PBR4 (sample PBR4_19).

**FIGURE 7:**
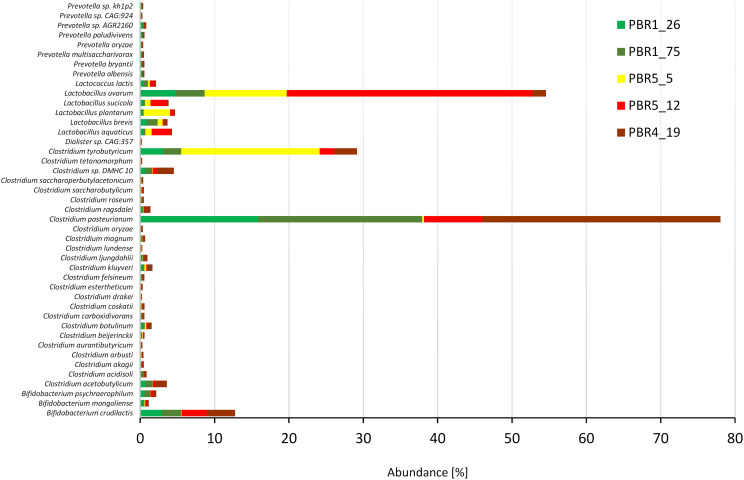
Most dominant species (abundance >0.1%) in selected samples from PBRs based on metagenomics analysis.

The 43 most dominant species with an abundance >0.1% belonged to six genera: *Clostridium* (25 species with *C. pasteurianum, C. tyrobutyricum, C. acetobutylicum*, *C. kluyveri, Clostridium* sp. DMHC 10), *Lactobacillus* (5 species with *L. uvarum, L. plantarum, L. aquaticus, L. brevis* and *L. sucicola*), *Bifidobacterium* (3 species with *B. crudilactis*), *Prevotella* (8 species) as well as *Lactococcus lactis* and *Dialister* sp. CAG:357. Strong quantitative differences between the contributions of *Clostridium* and *Lactobacillus* species in the bioreactors with different efficiencies of hydrogen production was also confirmed (Figure 7). The ratios *Clostridium*:*Lactobacillus* in the PBRs producing more than 100 cm^3^ hydrogen/g COD of molasses, PBR1 and PBR5 (sample PBR5_5) were 3:1 and 5:6, respectively, whereas in the PBR5 (PBR5_12) and PBR4 (PBR4_19) producing less than 50 cm^3^ hydrogen/g COD of molasses, they were 1:4 and 10:1, respectively. Interestingly, *C. pasteurianum* most abundant in PBR1 was replaced by *C. tyrobutyricum* in PBR5 during the period of its hydrogen production above 100 cm^3^ hydrogen/g COD of molasses. The ratios calculated based on metanogenic data show a comparable tendencies to those calculated based on 16S rRNA amplicons. They are compared with the results of other studies (Table 3) and discussed in the “Discussion” section. The contribution of *B. crudilactis* in all bioreactors was comparable and *L. uvarum* was the most abundant *Lactobacillus* species.

**TABLE 3:** A set of different studies presenting contribution of and proportions between hydrogen producing bacteria and lactic acid bacteria in hydrogen-producing microbial communities.

Lp.	Object of the study	Methods	Proportions between hydrogen producing bacteria and lactic acid bacteria	References
**1.**	Fermentation of glucose in agitated granular sludge bed bioreactors by the sludge from domestic wastewater treatment plant in a continuous system	Polymerase-chain reaction-denaturated gradient gel electrophoresis (PCR DGGE)	High efficiency of H_2_ production, contribution of *Clostridium* sp. and *Streptococcus* sp. was 68% and 26%, respectively, giving the ratio of HPB:LAB ≈ 2:1	Hung et al. (2007)
**2.**	Fermentation of cheese whey wastewater by anaerobic digester sludge in continuous system	Cloning and sequencing of the 16S rDNA gene amplified on the total DNA isolated from the microbial community	High efficiency of H_2_ production, *Lactobacillus* 50% (including 23% sequences similar to an unpublished highly efficient hydrogen-producing bacterium), *Clostridium* 9% and *Prevotella* 7%. The estimated ratio of HPB to LAB equals 39:27 ≈ 4:3	Yang et al. (2007)
**3.**	20 different lab-scale bioreactors with different configurations, operation conditions, and performances.	16S rRNA genes 454 pyrosequencing	High efficiency of H_2_ production. HPB:LAB ≈ 4:3, *Clostridium*:LAB ≈ 7:8; Medium efficiency of H_2_ production, HPB:LAB ≈ 1:1, *Clostridium*:LAB ≈ 1:2; Low efficiency of H_2_ production, HPB and LAB in minority, microbial communities dominated by the *Veillonellaceae* family	Etchebehere et al. (2016)
**4.**	Fermentation of tequila vinasse and Nejayote (from the maize industry) by microbial community from anaerobic digester treating food waste in batch bioreactor	Illumina MiSeq 16S rRNA sequencing	High efficiency of H_2_ production, *Lactobacillus casei, Clostridium beijerinckii, Acetobacter lovaniensis*, *Sporolactobacillus terrae* constituted 22.0%, 20.3%, 13.4% and 13%, respectively. *Clostridium*:LAB ≈ 2:3, *Clostridium*:*Lactobacillus* ≈ 1:1.	García-Depraect et al. (2017)
**5.**	Fermentation of sugarcane vinasse in continuously operating packed-bed reactor under thermophilic condition	Illumina MiSeq 16S rRNA sequencing	High efficiency of H_2_ production, *Clostridium* (16%):*Lactobacillus* (24%) ≈ 2:3, additionally the *Thermoanaerobacterium* genus was found as hydrogen producer.	Fuess et al. (2018)
**6.**	Fermentation of cheese whey powder with lactose by microbial community dominated by *Clostridium* and *Streptococcus* in continuously operating bioreactor	Illumina MiSeq 16S rRNA sequencing	High efficiency of H_2_ production, *Clostridium* (66%)*:Streptococcus* (28%) ≈ 2:1, Low efficiency of H_2_ production, *Clostridium* (38%)*:Streptococcus* (60%) ≈ 2:3	Palomo-Briones et al. (2018)
**7.**	Fermentation of tequila vinase in batch system by consortium (PTA-124566, American Type Culture Collection)	Illumina MiSeq 16S rRNA sequencing	Optimal H_2_ production, HPB (68.8%):LAB (15.2%):acetate producers (9.5%) ≈ 7:1.5:1	García-Depraect and León-Becerril (2018)
**8.**	Cofermentation of tequila vinasse and nixtamalization wastewater by the microbial community described in García-Depraect et al. (2017)	Illumina MiSeq 16S rRNA sequencing	High efficiency of H_2_ production, *Clostridium* (50.3%):*Lactobacillus* (12.0%), *Streptococcus* (3.8%) and *Sporolactobacillus* (2.7%) giving the ratio HPB:LAB ≈ 5:2	García-Depraect et al. (2019b)
**9.**	Cofermentation of tequila vinasse and nixtamalization wastewater by the microbial community described in García-Depraect et al. (2017)	Illumina MiSeq 16S rRNA sequencing	Optimal H_2_ production, *Clostridium* (55%):*Lactobacillus* (10%) and *Sporolactobacillus* (34%), giving the ratio HPB:LAB ≈ 5:4	Hung et al. (2007), García-Depraect et al. (2019c)
**10.**	Fermentation of tequila vinasse by bacterial consortium (ATCC PTA-124566) in batch experiments in well-mixed reactor	Illumina MiSeq 16S rRNA sequencing	Optimal H_2_ production, *Clostridium* (31%):*Lactobacillus* (55%) ≈ 1:2	Diaz-Cruces et al. (2020)
**11.**	Fermentation of molasses by the selected microbial communities in continuously operating PBRs	Illumina MiSeq 16S rRNA sequencing Illumina NextSeq metagenome sequencing	See Table 2 HPB (*Clostridium, Ruminococcus, Prevotella*):LAB (*Lactobacillus, Bifidobacterium, Leuconostoc, Streptococcus, Lactococcus*): High efficiency of H_2_ production, 2:1 (PBR1) or 1:2 (PBR5) Low efficiency of H_2_ production, 1:4 (PBR5) or 5:1 (PBR4) *Clostridium:Lactobacillus* High efficiency of H_2_ production, 3:1 (PBR1) or 5:6 (PBR5) Low efficiency of H_2_ production, 1:4 (PBR5) or 10:1 (PBR4)	This study

**FIGURE 8:**
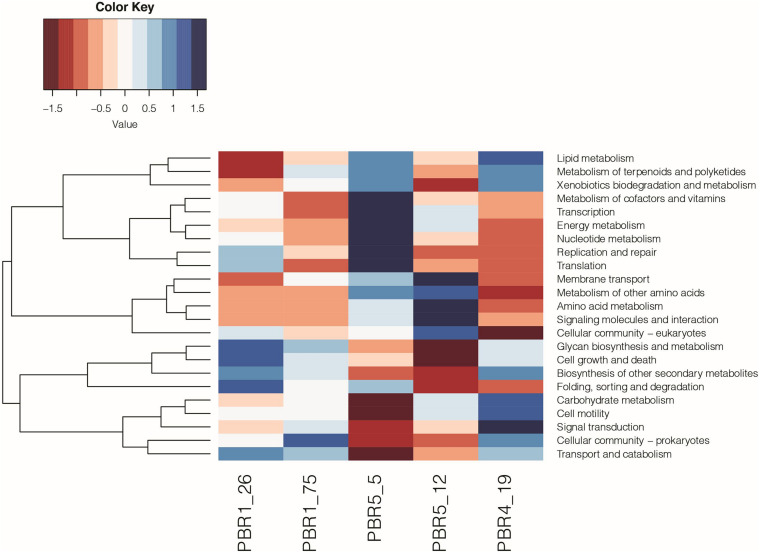
Metabolic potential based on KEGG (level 2) analysis of the microbial communities selected out in PBR1 (PBR1_26, PBR1_75), PBR5 (PBR5_5, PBR5_12) and PBR4 (PBR4_19).

The functional analysis (KEGG analysis) revealed differences between microbial communities that selected out in bioreactors PBR1, PBR4 and PBR5 (Figure 8 and Supplementary Table 8). In comparison to the PBR4 and PBR5 the microbial communities collected from the PBR1 bioreactor (samples PBR1_26 and PBR1_75) show underrepresentation of the pathways of lipid metabolism, energy metabolism, amino acids metabolism, and signaling molecules and interaction, whereas the pathways of transport and catabolism, glycan biosynthesis and metabolism, biosynthesis of other secondary metabolites, cell growth and death are overrepresented. A comparable metabolic potential observed for samples PBR1_26 and PBR1_75 collected within 49 days of each other confirms a stable hydrogen production (>100 cm^3^/g COD of molasses) in PBR1. Little resemblance between metabolic potential of the microbial community in PBR1 and PBR5 at the beginning of its operation (sample PBR5_5 with hydrogen production >100 cm^3^/g COD of molasses) can be associated with different operational conditions for both bioreactors, such as HRT, working volume of the bioreactor, conditions for biofilm and granules formation (lack of bioreactor filling bed in PBR5), HRT, inoculum (for details see Table 1). An interesting finding is the overrepresentation of folding, sorting and degradation pathways in bioreactors with hydrogen production >100 cm^3^/g COD molasses (samples PBR1_26, PBR1_75, PBR5_5) and their underrepresentation in those with hydrogen production <50 cm^3^/g COD molasses (samples PBR5_12 and PBR4_19). Decrease and instability of hydrogen production in PBR5 was associated with changes in metabolic potential (sample PBR5_5 vs. sample PBR5_19), especially the carbohydrates metabolism. Noteworthy is the contrast between samples PBR5_5 and PBR4_19. Direction of the changes in almost all of the pathways analyzed is opposite, except for the lipid metabolism, the xenobiotics biodegradation and metabolism, and the metabolism of terpenoids and polyketides.

## Discussion

### Dynamics and Metabolic Activity of DF Microbial Communities

Here, we provided new data on the dynamics and the metabolic activity of DF microbial communities in relation to the efficiency of hydrogen production. The study involved tracking the continuous operation of five hydrogen-producing PBRs and examining the microbial communities by 16S rDNA profiling and further metagenomics analysis of the selected consortia. The results are discussed taking into account the knowledge of the problems associated with the instability of hydrogen production.

As it has been highlighted in a recent excellent review paper (Castelló et al., 2020), hydrogen-producing microbial communities show (i) a relatively low diversity in comparison to methanogenic communities and (ii) tendencies to change, which is reflected in the instability of hydrogen production. It is thought that microbial communities of low diversity are less stable and have limited rebuilding capabilities. Those theses are supported by our study. First, the microbial communities producing more than 100 cm^3^ of hydrogen per g COD of molasses that selected out in PBR1 are the most stable in time and show the highest biodiversity measured by taxonomic richness and evenness. Secondly, the differences observed in the composition of the microbial communities with unstable hydrogen production are not related to the emergence of new taxa of bacteria. The problem stems from changes in the proportions between specific taxa, especially the HPB and the LAB.

### Lactic Acid Bacteria in DF Microbial Communities

Extremely intriguing is the problem of the presence of the LAB and their ambiguous role (negative or positive) in the hydrogen-producing microbial communities. Our observations regarding the low efficiency of hydrogen production in PBR4 operation at the point of PBR4_5 sample collection and PBR5 operation at the point of PBR5_12 sample collection confirm the commonly recognized fact about the negative role of the LAB in DF bioreactors. They are regarded as microorganisms that compete for the substrate and shift the type of fermentation toward lactic acid fermentation (Noike et al., 2002; Ren et al., 2007; Jo et al., 2008; Sreela-Or et al., 2011; Etchebehere et al., 2016; Palomo-Briones et al., 2018). Analysis of the non-gaseous fermentation products in PBR4_5 and PBR5_12 samples revealed lactic acid as the dominant fermentation product and a low concentration of butyric acid. Furthermore, noteworthy was the high concentration of ethanol in the PBR4_5 sample and, interestingly, in the PBR5_5 sample. The latter was a sample collected during the efficient hydrogen production by PBR5, but shortly before the period of decrease in the hydrogen production (Table 1). In our previous studies on inhibition of hydrogen fermentation by yeasts we also observed a significant increase in the concentration of ethanol in the effluent. The other changes were similar to observed in the microbial communities dominated by LAB, i.e., an increase in lactate, decrease in butyrate and a drop in pH to <4.0 (Detman et al., 2018). Here, the presence of yeasts was excluded based on regular microscopic image monitoring. Thus concentration of ethanol might be a relevant marker or factor promoting a metabolic shift in the DF bioreactors processing carbohydrates toward solventogenic pathways. However, in a study of Ren et al. (2007) on molasses fermentation it was shown that hydrogen was produced most efficiently in the ethanol-type fermentation at the pH 4.0–4.5 by a microbial community dominated by the HPB affiliated with the *Ethanoligenens* genus. In our experiments metagenomics revealed *Ethanoligenens harbinense* as a minor genus in the examined PBRs (0.2–0.3% in PBR1; <0.01% in PBR4 and PBR5).

Our results clearly show that proportions between LAB and HPB are key for the processes of hydrogen production. A multiple predominance of LAB over HPB or *Clostridiaceae* over LAB results in decreased hydrogen production. Under the optimal conditions for hydrogen production (PBR1) the most abundant taxons in the microbial communities were: *Clostridiaceae* with the dominant genus *Clostridium* (*C. pasteurianum, C. tyrobutyricum, C. kluyveri, C. acetobutylicum*), *Lactobacillaceae* with *Lactobacillus* (*L. uvarum, L. plantarum, L. aquaticus, L. sucicola, L. brevis*), *Ruminococcaceae*, *Prevotellaceae* with *Prevotella* and *Bifidobacteriaceae* with *Bifidobacterium*. Many studies describing optimal conditions for hydrogen production and analyzing structures of the respective DF microbial communities (set out in Table 3) clearly show similar tendencies, especially regarding the contribution of LAB, HPB and additionally acetate-producers (Hung et al., 2007; Yang et al., 2007; Etchebehere et al., 2016; García-Depraect et al., 2017, 2019a, 2019b, 2019c; García-Depraect and León-Becerril, 2018; Palomo-Briones et al., 2018; Fuess et al., 2019; Diaz-Cruces et al., 2020). The balance between putative HPB and LAB with the tendency for the former to prevail is typical for the most optimal conditions of hydrogen production in the majority of cases. A significant dominance of one group over another disturbs the hydrogen production process. There are common trends observed independent of the experimental set-ups and techniques used for analysis of the microbial communities. However, it should be remembered that such factors as type of feedstock or operating conditions are specific for the individual research setups and should be considered when investigating and optimizing the performance of dark fermentation bioreactors.

The PBRs producing hydrogen at the rate >100 cm^3^ per g COD of molasses clearly exhibit a low concentration of lactic acid in the non-gaseous fermentation products despite the presence of LAB in the microbial communities. It is in accordance with our previous results (Chojnacka et al., 2011; Detman et al., 2017) and indicates cross-feeding of lactate in bioreactors. Our previous study confirmed that DF microbial communities fermenting molasses convert lactate and acetate to butyrate (Detman et al., 2019). Recently, we have reported that the balance between LAB and butyrate-producing clostridia, and the pH conditions are the most relevant factors for the process of conversion of lactate and acetate to butyrate. Furthermore, the structure of the microbial communities most efficiently producing hydrogen presented here is comparable to the structure of consortia capable of conversion of lactate and acetate to butyrate (Detman et al., 2020). This supports the significance of the lactate and acetate conversion pathways in hydrogen-producing microbial communities postulated by other authors (Hung et al., 2007; Matsumoto and Nishimura, 2007; Yang et al., 2007; Jo et al., 2008; Juang et al., 2011; Wu et al., 2012; García-Depraect et al., 2017, 2019a,b, 2019c; García-Depraect and León-Becerril, 2018; Palomo-Briones et al., 2018; Fuess et al., 2019).

The difference in our study consisting a relatively significant abundance of *Caproiciproducens* reflects the methodology used (analysis of 16S rRNA amplicons and metagenomic data analysis).

Previously we postulated that pH is a key factor responsible for the balance of DF microbial communities (Chojnacka et al., 2011; Sikora et al., 2013). Studies by other authors clearly showed that pH in the range of 5.5–6.5 was optimal for butyrate, hydrogen and carbon dioxide production from lactate and acetate (Matsumoto and Nishimura, 2007; Jo et al., 2008; Juang et al., 2011; Wu et al., 2012; Palomo-Briones et al., 2018; Fuess et al., 2019; García-Depraect et al., 2019b). Interestingly, the pH of the effluents from all PBRs was comparable independent of the hydrogen production efficiency. However, local pH increases inside PBRs cannot be excluded since the microbial community forms different structures such as granules and biofilm (Chojnacka et al., 2011).

### Product Inhibition in Hydrogen-Producing PBRs

PBR4, PBR5, and PBR3 shifted toward the solvatogenic pathway characterized by overproduction of non-gaseous fermentation products and a dramatic drop in the hydrogen yield. The maximum hydrogen yield during clostridial-type fermentation is 4 moles of hydrogen per mole of glucose when the only other products are acetic acid and carbon dioxide (Hallenbeck, 2009). It is commonly recognized that when the hydrogen partial pressure increases, the activity of NFOR is inhibited. NFOR catalyzes the formation of reduced ferredoxin in the reaction with NADH (NADH + Fd → NAD^+^ + FdH). The excess hydrogen is used in the synthesis of short-chain fatty acids and alcohols, which promotes a metabolic shift and a pH drop in the bioreactor being unfavorable for bio-hydrogen production. To prevent hydrogen accumulation, methods are employed to accelerate hydrogen release (Hallenbeck and Ghosh, 2009; Elbeshbishy et al., 2017). Inhibition of hydrogen production by overproduction of SCFAs is well illustrated by the sample PBR4_19 where in the microbial community the *Clostridium* (53.8%) and *Prevotella* (5.5%) genera overwhelmed five-fold the sum of *Bifidobacterium* (5.4%) and *Lactobacillus* (5.4%). The analysis of most abundant species showed a 10-fold predominance of the *Clostridium* species over the *Lactobacillus* species. This is another evidence that abundance of *Clostridiaceae* in the DF microbial community is not optimal for hydrogen production and the LAB are a relevant component of microbial communities producing hydrogen most effectively.

Recently, it has been shown that production of hydrogen or SCFAs and alcohols during dark fermentation can be controlled by redox mediators that change the redox potential and the electron transfer between enzymatic complexes and drive NADH toward reactions leading to hydrogen formation (Atilano-Camino et al., 2020). Furthermore, it was found that *C. pasteurianum* changed its metabolites from acetate, hydrogen, carbon dioxide and butyrate to lactate, ethanol and butanol under the conditions of low pH and iron and phosphate deficit (Dabrock et al., 1992). Ferredoxins and hydrogenases contain iron–sulfur (Fe–S) clusters in their active sites whose role is electron transport (Das et al., 2006).

### Other Factors Relevant for Hydrogen Production

One of the problems concerning the instability of hydrogen production is the presence of propionate fermenters and methanogenic archaeons that consume hydrogen. In our system methane has never been detected, neither in this study nor previously (Chojnacka et al., 2011; Detman et al., 2017). Also, concentrations of propionate were relatively low in the samples of effluents examined. This indicates that propionate-type fermentations characteristic of, e.g., *Clostridium propionium* (Baghchehsaraee et al., 2009) or *Blautia* and *Propionicum* genera (García-Depraect et al., 2019b) were irrelevant in the PBRs. The exception was sample PBR3_12 where processes of solvatogenesis were increased.

Symbiotic interactions between bacteria resulting from proper proportions between bacteria are determined by (i) the source of microorganisms and the selection it undergoes; (ii) operating conditions such as bioreactor construction, packing material and substrate concentration that are crucial for the continuous, stable process of hydrogen production (Castelló et al., 2020).

Analysis of the performance of PBRs indicates great importance of the source of the inoculum. Long-term selection and specialization of the microbial community fed on the same substrate ensures stabilization of the hydrogen production process. The stabilization and long-term performance are maintained by the operating conditions. A very important factor for hydrogen production is a proper bioreactor construction enabling the retention of biomass (Chojnacka et al., 2011; Detman et al., 2017). This is achieved by the use of a packing material constituting a support for biofilm and granules formation. Unstable performance and changes in the microbial community over a short time observed in PBR5 may result from the lack of packing material in this reactor. On the other hand, continuous operation of PBRs requires regular removal of the excess of bacterial biomass that inhibits hydrogen production and promotes solvatogenesis. Overproduction of non-gaseous fermentation products and biomass formation are favored by a higher substrate concentration. It is illustrated by PBR3 (34 g COD of molasses/L), where the development of all groups of bacteria and a large variety of non-gaseous fermentation products were observed. On the other hand, a decrease of substrate concentration to 8.5 g COD of molasses/L inhibited the development of LAB in the microbial community (PBR2) that supports our current (PBR4) and previous (Detman et al., 2020) observations that sucrose stimulates LAB in DF consortia. Other factors preventing biomass accumulation and overproduction of SCFAs or alcohols are the HRT and the working volume that, together with the proper substrate concentration, ensure effective substrate utilization and gas release (Castelló et al., 2020).

Fulfillment of the conditions for good performance, as observed for PBR1 operation, ensures balance and stability of the microbial community.

## Conclusion

Instability of hydrogen production was closely related to the changes in the non-gaseous fermentation products and the composition of the microbial communities. Increased concentration of ethanol might be a relevant marker or factor promoting a metabolic shift from hydrogen-yielding fermentation toward overproduction of non-gaseous fermentation products (solventogenic pathways). Further investigations are required.

The microbial community producing above 100 cm^3^ of hydrogen per g COD of molasses showed the highest biodiversity and stability. Instability of hydrogen production was related to the changes in the proportions between specific taxa, especially the HPB and the LAB, rather than to the emergence of new taxa of bacteria. The highest efficiency of hydrogen production (PBR1) was achieved at the ratios of HPB to LAB of 4:2.5 or 2:1, as determined by 16S rRNA sequencing or metagenomics, respectively. The identified HPB were *Clostridium*, especially *C. pasteurianum* and *C. tyrobutyricum*, *Ruminococcus* and *Prevotella*, the identified LAB were *Lactobacillus* especially *L. uvarum*, *Bifidobacterium, Leuconostoc, Streptococcus, Lactococcus*.

Dominance of one group over another disturbs the hydrogen production process. The lowest efficiency of hydrogen production was associated with a three-fold to four-fold prevalence of LAB over HPB or 5- to 60-fold prevalence of clostridia over LAB, as determined by both 16S rRNA sequencing and metagenomics.

We have compared our results with those of other research groups and found that under the conditions optimal for hydrogen production a specific balance between hydrogen producers and LAB was established with a tendency for hydrogen producers to prevail.

Interestingly, analysis of metabolic potential of the microbial communities revealed overrepresentation of folding, sorting and degradation pathways in bioreactors with hydrogen production >100 cm^3^/g COD of molasses and their underrepresentation in those with hydrogen production <50 cm^3^/g COD of molasses.

Identifying a community balance between hydrogen and lactic acid producers is crucial for obtaining stable hydrogen producing systems and their optimization. The source of inoculum and its selection, operating conditions such as bioreactor construction, packing material, substrate concentration and hydraulic retention time, are key factors that help maintain an optimal equilibrium inside the microbial community and warrant a stable process of hydrogen production.

## Data Availability Statement

The datasets presented in this study can be found in online repositories. The names of the repository/repositories and accession number(s) can be found in the article and Supplementary Material.

## Author Contributions

AD planned the work, conceived and designed the experiments, isolated microbial DNA, analyzed the results, and contributed to writing the manuscript. DL performed DNA sequencing (16S rRNA and metagenomics), analyzed the sequence data, and contributed to writing the manuscript. AC conceived and designed the experiments, and isolated microbial DNA. EW-S collected the data describing PBRs’ performance. JP coordinated the project and supplied financial support. ASa performed analyses of short chain fatty acids and ethanol. WK constructed the PBRs. MB revised the manuscript. AB, YC, and FY analyzed the metagenomics data. EŁ performed statistical analyses of PBRs performance. ASi planned the work, conceived and designed the experiments, analyzed the data, and wrote the manuscript. All authors read and approved the final manuscript.

## Conflict of Interest

EW-S and JP were employed by company Krajowa Spółka Cukrowa S.A. “Polski Cukier”. The remaining authors declare that the research was conducted in the absence of any commercial or financial relationships that could be construed as a potential conflict of interest.

## Footnotes

1 https://github.com/yhwu/idemp

2 https://ggplot2.tidyverse.org/

3 https://www.kegg.jp/ghostkoala/

## Supplementary Material

Supplementary Figure 1 Alpha Diversity (a. Shannon, b. Simpson) of the microbial communities that selected out in PBRs.

Supplementary Figure 2 Taxonomic composition of the microbial communities from the selected samples based on metagenomics analysis: a. phylum level, b. class level, c. family level, d. genus level.

Supplementary Figure 3 Relative abundances of the dominant phyla derived from metagenomic sequencing and 16S rRNA amplicon sequencing of the selected samples (PBR1_26, PBR1_75, PBR5_5, PBR5_12 and PBR4_19).

Supplementary Table 1 Over time analysis of hydrogen production in PBR1 (Kolmogorov–Smirnov and Mann–Whitney tests).

Supplementary Table 2 Over time analysis of hydrogen production in PBR2 (Kolmogorov–Smirnov and Mann–Whitney tests).

Supplementary Table 3 Over time analysis of hydrogen production in PBR3 (Kolmogorov–Smirnov and Mann–Whitney tests).

Supplementary Table 4 Over time analysis of hydrogen production in PBR4 (Kolmogorov–Smirnov and Mann–Whitney tests).

Supplementary Table 5 Over time analysis of hydrogen production in PBR5 (Kolmogorov–Smirnov and Mann–Whitney tests).

Supplementary Table 6 Detailed taxonomic composition (genus level) of the microbial communities that selected out in PBRs, based on hypervariable V4 region of the 16S rRNA gene, sequenced on MiSeq platform (Illumina). The taxonomy was assigned using RDP classifier against the SILVA database.

Supplementary Table 7 Detailed taxonomic composition of the microbial communities from the selected samples based on metagenomics analysis: phylum, class, order, family, genus, species.

Supplementary Table 8 Detailed data describing metabolic potential based on KEGG (level 2) analysis of the selected microbial communities (PBR1_26, PBR1_75, PBR5_5, PBR5_12 and PBR4_19) presented in Figure 8.
