# Supplementary figures and images for: Dynamics and Complexity of Dark Fermentation Microbial Communities Producing Hydrogen From Sugar Beet Molasses in Continuously Operating Packed Bed Reactors
Source: Front Microbiol. 2021 Jan 8;11:612344. doi: 10.3389/fmicb.2020.612344 (PMC7819888; doi:10.3389/fmicb.2020.612344)

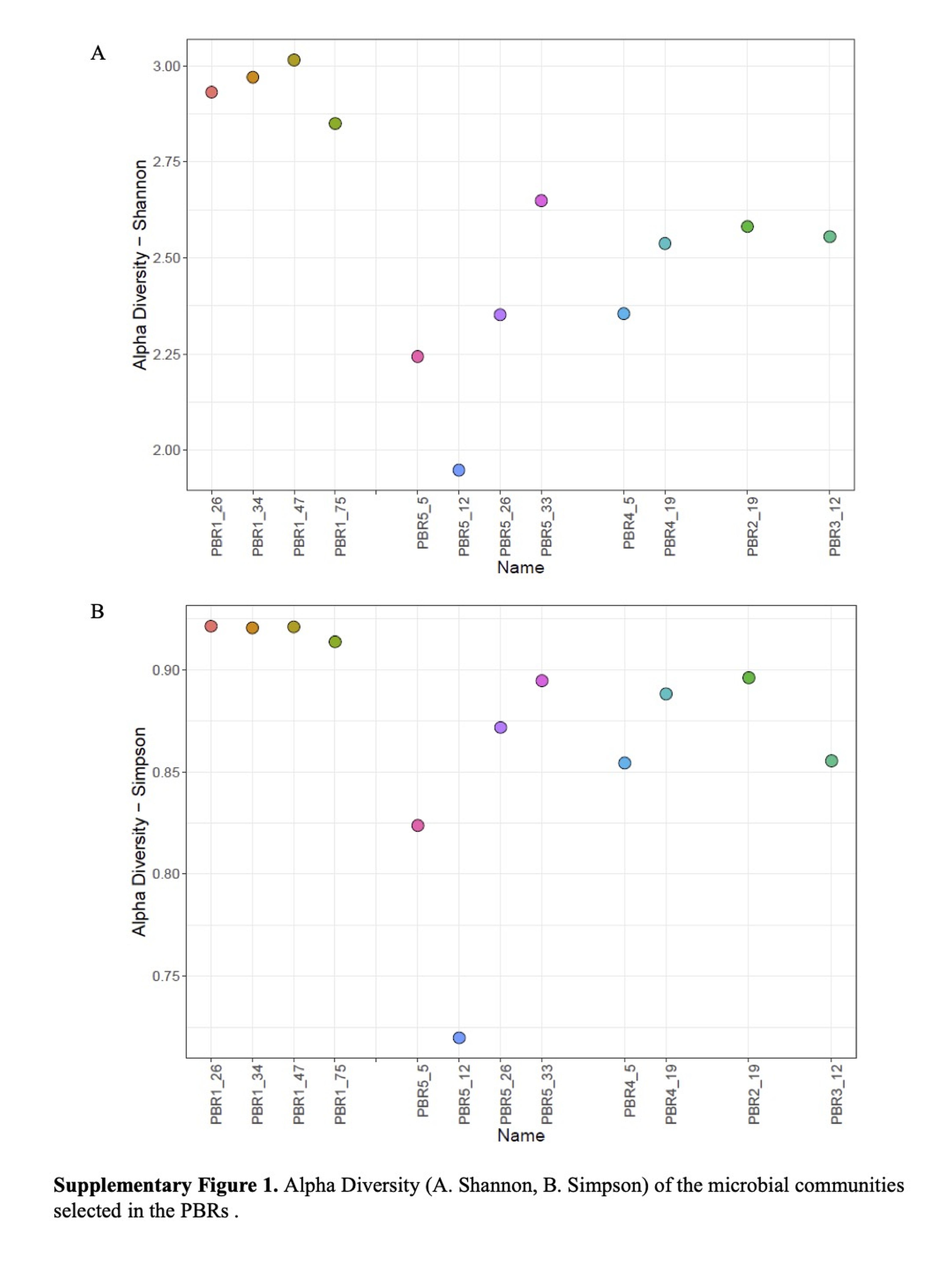

Supplement: Supplementary Figure 1 — Alpha Diversity (a. Shannon, b. Simpson) of the microbial communities that selected out in PBRs. [file Image_1.jpg]

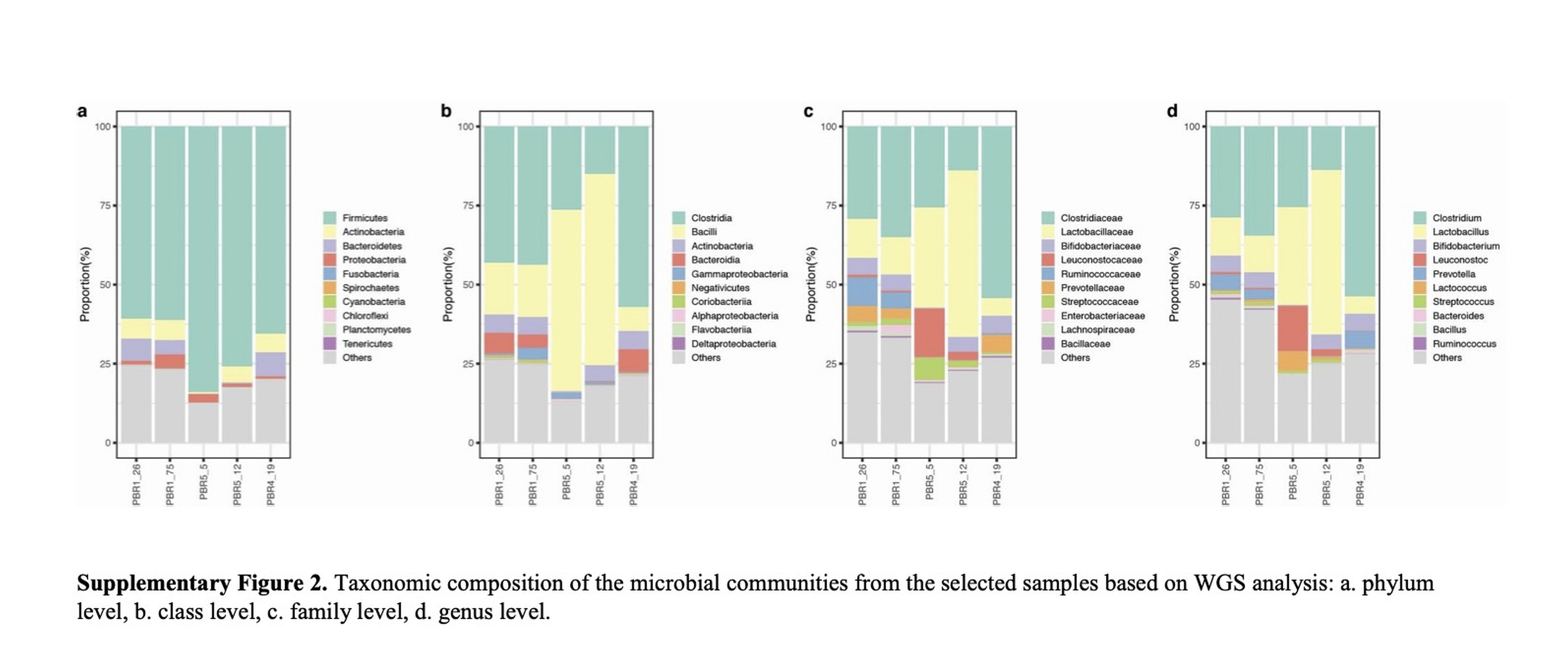

Supplement: Supplementary Figure 2 — Taxonomic composition of the microbial communities from the selected samples based on metagenomics analysis: a. phylum level, b. class level, c. family level, d. genus level. [file Image_2.jpg]

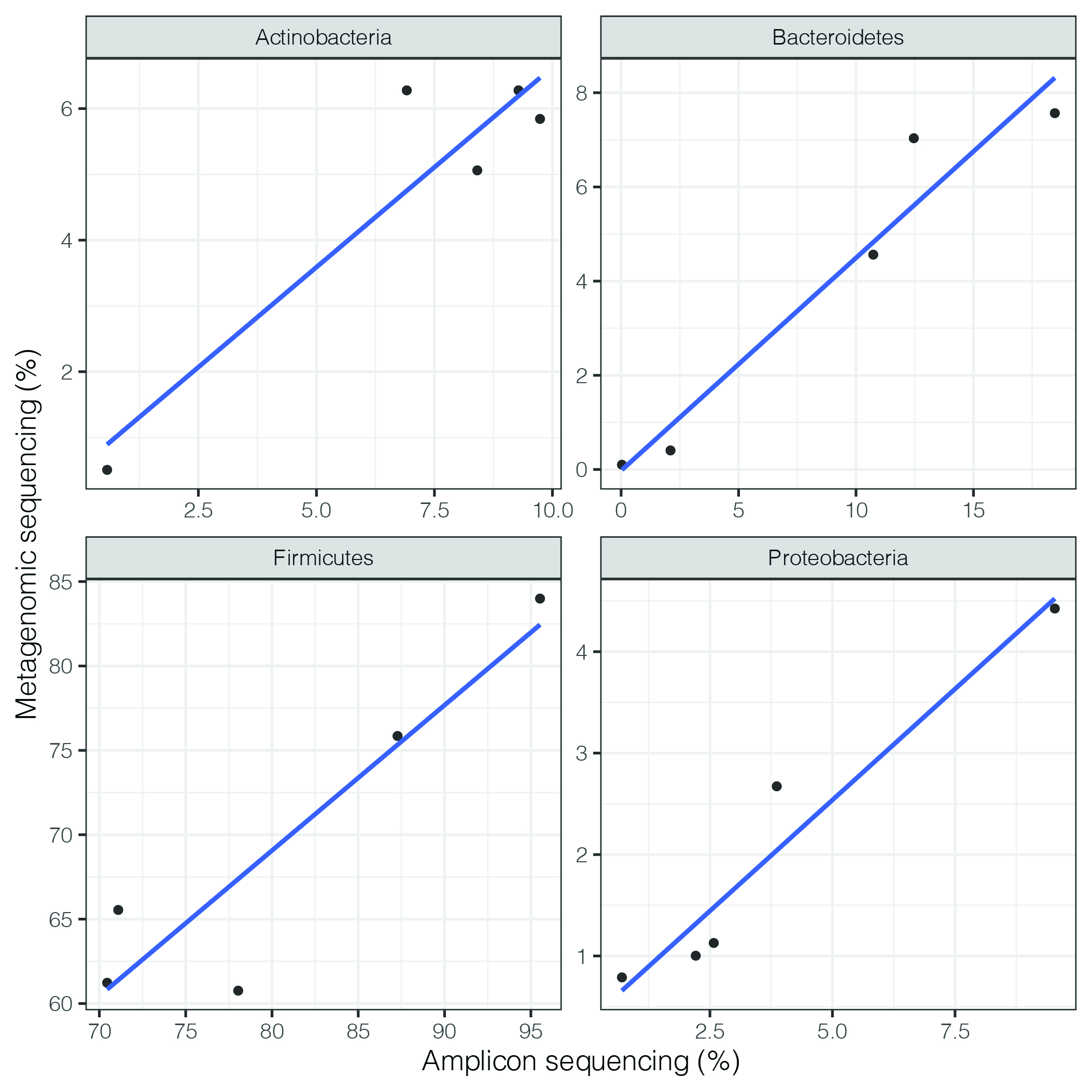

Supplement: Supplementary Figure 3 — Relative abundances of the dominant phyla derived from metagenomic sequencing and 16S rRNA amplicon sequencing of the selected samples (PBR1_26, PBR1_75, PBR5_5, PBR5_12 and PBR4_19). [file Image_3.jpg]
